# A Systematic Review on the Application of Virtual Reality for Muscular Dystrophy Rehabilitation: Motor Learning Benefits

**DOI:** 10.3390/life14070790

**Published:** 2024-06-22

**Authors:** Pawel Kiper, Sara Federico, Joanna Szczepańska-Gieracha, Patryk Szary, Adam Wrzeciono, Justyna Mazurek, Carlos Luque-Moreno, Aleksandra Kiper, Mattia Spagna, Rita Barresi, Błażej Cieślik

**Affiliations:** 1 Healthcare Innovation Technology Lab, IRCCS San Camillo Hospital, 30126 Venezia, Italy; sara.federico@hsancamillo.it (S.F.); blazej.cieslik@hsancamillo.it (B.C.); 2 Faculty of Physiotherapy, Wroclaw University of Health and Sport Sciences, 51-612 Wroclaw, Poland; joanna.szczepanska@awf.wroc.pl (J.S.-G.); patryk.szary94@gmail.com (P.S.); awp97adam@wp.pl (A.W.); 3 University Rehabilitation Centre, Wroclaw Medical University, 50-367 Wroclaw, Poland; justyna.mazurek@umed.wroc.pl; 4 Department of Physical Therapy, Faculty of Nursing, Physiotherapy and Podiatry, University of Seville, 41009 Seville, Spain; carloslm@us.es; 5 Institute of Biomedicine of Seville (IBIS), 41013 Seville, Spain; 6 Doctoral School of the University of Rzeszów, University of Rzeszów, 35-959 Rzeszów, Poland; akiper94@gmail.com; 7 Neurobiology Lab, IRCCS San Camillo Hospital, 30126 Venezia, Italy; spagnamattia97@gmail.com (M.S.); rita.barresi@hsancamillo.it (R.B.)

**Keywords:** muscular dystrophy, virtual reality, myotonic dystrophy, neuromuscular, myopathy, physical therapy, exergaming, motion capture

## Abstract

Using virtual reality (VR) for Muscular Dystrophy (MD) rehabilitation promises to be a novel therapeutic approach, potentially enhancing motor learning, functional outcomes, and overall quality of life. This systematic review primarily aimed to provide a comprehensive summary of the current understanding regarding the application of VR in supporting MD rehabilitation. A systematic search was performed in PubMed, Scopus, Cochrane Library, and Web of Science to identify relevant articles. The inclusion criteria encompassed studies involving individuals diagnosed with MD who underwent VR interventions, with a primary focus on assessing functional improvement. Methodological quality of the studies was assessed by using the Physiotherapy Evidence Database (PEDro) scale. Seven studies, involving 440 individuals with Duchenne Muscular Dystrophy (DMD), were included in the review. Among these studies, six primarily explored the motor learning potential of VR, while one study investigated the impact of VR training on functional abilities. In conclusion, the qualitative synthesis supports VR-based interventions’ potential positive effects on motor learning, performance improvement, and functional outcomes in individuals with DMD. However, current usage mainly focuses on assessing the potential mechanisms’ benefits, suggesting the importance of expanding clinical adoption to harness their therapeutic potential for MD patients.

## 1. Introduction

Muscular dystrophy (MD) encompasses a heterogeneous group of genetic disorders characterized by dystrophic changes in muscle structure [1]. The inheritance patterns include autosomal dominant, autosomal recessive, or X-linked transmission, with occasional cases arising from de novo mutations [2]. Typically, disease severity correlates with the age of onset, with earlier manifestation leading to more severe symptoms. Clinical presentations vary depending on the specific type of muscular dystrophy, with common symptoms such as skeletal muscle function loss, joint contracture, scoliosis, and osteoporosis [3].

Its global incidence varies according to the specific MD type [4]. The diagnosis of MD involves clinical evaluation, electromyographic examination (EMG), and muscle biopsy. Advances in molecular genetics have enabled the identification of over 50 distinct forms of MD, including Duchenne muscular dystrophy (DMD) and Becker muscular dystrophy, myotonic dystrophy, facial-scapulo-brachial dystrophy, limb-girdle muscular dystrophy (LGMD), congenital muscular dystrophy, Emery–Dreifuss dystrophy, among others [5]. DMD stands as the most prevalent form of MD, primarily inherited as an X-linked recessive disorder affecting males. Nevertheless, isolated cases have been reported in females with Turner syndrome. DMD is distinguished by the absence of dystrophin protein in the muscle fiber membrane, stemming from a mutation in the Xp21 gene [5]. Its frequency is approximately 1 in every 3500 males [6].

Virtual reality (VR) has gained significant prominence in clinical rehabilitation due to its extensive utilization for both assessment and training of various disorders [7,8,9]. The primary aim of VR is to create an immersive experience, defined as a state where users feel deeply engaged and mentally absorbed in the virtual environment, diminishing real-world sensations and fostering a heightened sense of presence within the virtual space [10]. Four distinct types of VR are recognized, as follows, each offering unique experiences: Non-immersive VR, often referred to as virtual desktop reality, operates solely using a computer, phone, or tablet screen [10]. Non-immersive VR includes motion capture technology, which records and replicates human movements within virtual environments, and exergaming, a form of interactive gaming that combines physical exercise with gameplay experiences to promote fitness and engagement [11]. In contrast, immersive VR isolates the participant completely from the real world, delivering information exclusively from a computer-generated environment and requiring specialized equipment such as VR goggles, motion controllers, or a body-map peaking camera [12]. The virtual environment offers numerous opportunities for visually representing repetitive movements, enabling rehabilitation through various VR scenarios [13,14]. This could be especially important in the context of motor learning, which, as the process through which individuals acquire and refine motor skills via practice and experience, is recognized as a fundamental aspect of rehabilitation [15]. Recent literature reviews have underscored the significance of motor learning mechanisms, highlighting principles and phenomena pivotal in shaping recovery patterns in neurological diseases [16]. Augmented feedback can be delivered in the virtual setting, utilizing a high-fidelity computer interface to provide real-time simulation and task-oriented feedback akin to real-world scenarios. As a result, VR-based motor training has emerged as a potent motor learning-based approach for effectively addressing diverse impairments.

Managing muscular dystrophy involves addressing a multitude of symptoms which significantly impact the patients’ quality of life [17]. While current rehabilitation strategies, such as physical and occupational therapy, are valuable, they are often constrained by their reliance on in-person therapy sessions and their limited ability to mimic real-world scenarios [18,19]. Moreover, motivation and engagement are critical factors in successful pediatric rehabilitation, and traditional methods may struggle to sustain the child’s interest over extended periods [20]. Virtual reality emerges as a potential solution to these challenges. By creating an engaging and dynamic environment, VR interventions can encourage consistent practice and may offer greater flexibility [21]. For individuals with MD, this is particularly important, as maintaining and improving functional abilities requires ongoing effort and commitment. VR technology could make this process more engaging and sustainable, potentially leading to better outcomes.

Despite the potential benefits of VR, the existing literature on its application and effectiveness in MD rehabilitation is both sparse and fragmented. To date, also, systematic reviews that have assessed the utilization of virtual reality technologies for the motor treatment of patients with dystrophy are limited as well [22]. This can be attributed to the rarity of muscular dystrophy and the absence of a definitive cure, limiting the scope for extensive research. Consequently, the consolidation of existing knowledge becomes crucial, as such a synthesis may shape future advancements in targeted therapies or rehabilitation strategies. Therefore, this study primarily aimed to provide a comprehensive summary of the current understanding regarding the application of virtual reality in supporting muscular dystrophy rehabilitation. Furthermore, this study sought to quantitatively assess the effectiveness of virtual reality in improving functional outcomes in comparison to standard care.

## 2. Materials and Methods

### 2.1. Design and Protocol Registration

This study was designed as a systematic review with meta-analysis. The protocol of this review was registered a priori with the PROSPERO database (CRD42020192761). The Preferred Reporting Items for Systematic Reviews and Meta-Analyses (PRISMA) Statement was followed for reporting [23].

### 2.2. Literature Search, Study Selection, and Data Extraction

A systematic article search was conducted in PubMed, Scopus, Cochrane Library, and Web of Science, encompassing the period up to 31 July 2023. Our objective was to include studies focusing on individuals diagnosed with MD who underwent virtual reality interventions. In this review, we selected articles that provided detailed information about the utilization of virtual reality systems in training programs and included comparisons to conventional treatment (CT), no intervention, placebo, or a combination of virtual reality with CT. The primary outcome of interest was the assessment of functional improvement in the patients. Secondary outcomes encompassed enhancements in gait, Activities of Daily Living (ADL), reductions in muscle fatigue, and increases in muscle strength. This systematic review comprised randomized clinical trials (RCT), quasi-RCT, and case-control studies. We excluded studies involving patients with multiple co-morbidities or other neurological impairments, as well as those utilizing robots as part of the intervention. Furthermore, the search was restricted to studies published in the English language to maintain consistency in the review.

To thoroughly search and identify pertinent articles in these databases, we employed a comprehensive set of keywords and their variations related to PICO. For participant criteria, we used terms such as “Muscular Dystrophies”, “Myotonic Dystrophy”, and “MD1”. Regarding the intervention, we included keywords like “virtual reality”, “exergaming”, and “VR”. As for the outcomes, we considered terms such as “motor treatment”, “muscle strength”, and “motor learning” (please refer the Supplementary Materials for the complete search strategy for each database).

The studies obtained through the search strategy, accompanied by the study information and abstract text, were imported into the Systematic Review Assistant-Deduplication Module for the purpose of de-duplication [24,25]. Subsequently, two reviewers conducted the screening process, evaluating both abstracts and full texts. Independently, they extracted relevant data based on pre-established inclusion criteria, utilizing Rayyan AI software [26]. In cases where discrepancies arose between the two reviewers’ opinions, a third reviewer served as a moderator to resolve any differences. We extracted data related to the study design, population characteristics, interventions and outcomes used, and the study conclusions.

### 2.3. Quality Assessment

The included studies were evaluated for methodological quality and risk of bias using the Physiotherapy Evidence Database (PEDro) scale. This scale comprises 11 yes-or-no questions that are scored, resulting in a methodological quality score. A score of 9 to 10 is considered excellent, 6 to 8 is good, 4 to 5 is fair, and 3 or below indicates poor quality [27]. Additionally, each study received an internal validity score (IVS), which was calculated by extracting seven specific PEDro items (items 2, 3, and 5 through 9) as suggested by van Tulder (23). The scores for these items were summed to obtain a collective IVS score. A value of 6–7 on the IVS is considered to be indicative of high methodological quality, 4–5 represents moderate methodological quality, and 0–3 points indicate limited methodological quality for the study [28,29].

## 3. Results

### 3.1. Result of the Search

**Figure 1:**
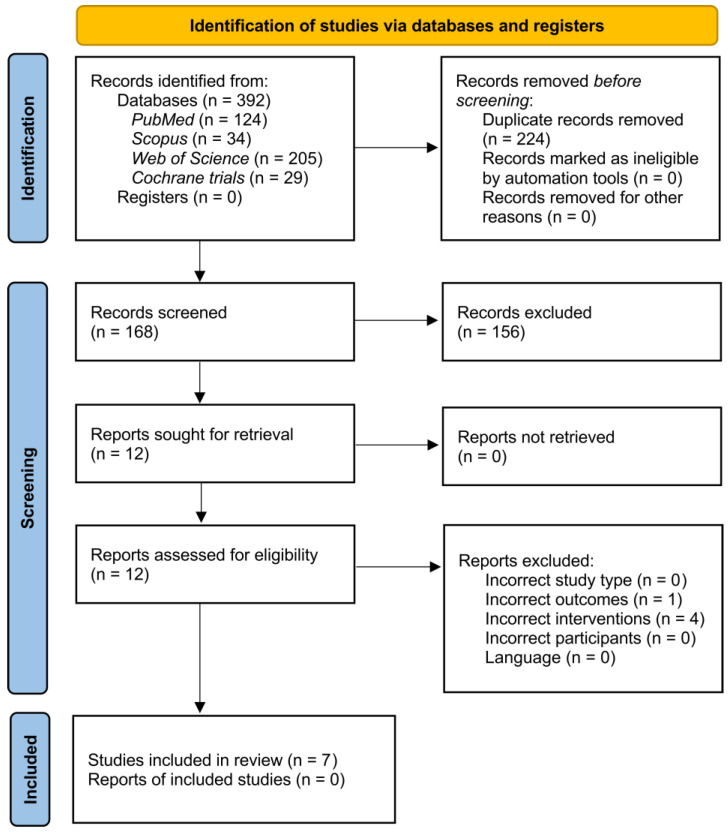
PRISMA study flow diagram.

The systematic search yielded 392 records, of which 168 remained after removing duplicates and screening titles and abstracts. Following the initial screening procedure, 12 articles were considered for the full-text review. After a full-text assessment, seven studies involving 440 participants were included in the qualitative synthesis. The flow of the study’s identification and selection process is summarized in Figure 1.

### 3.2. Characteristics of the Included Studies

Table 1 illustrates the characteristics of the included studies. All of the studies included in the review were primarily focused on using virtual reality rehabilitation approaches for individuals with Duchenne muscular dystrophy, specifically targeting the rehabilitation of the upper limbs. Among the seven included studies, six primarily investigated motor learning and movement mechanisms [30,31,32,33,34,35]. These six studies compared the motor learning patterns between individuals with DMD and typically developed (TD), while one study explored motor learning differences within the DMD group across different tasks. In five of the studies, the assessment of motor learning included measurements of acquisition, retention, and transfer, while the remaining study focused on evaluating the speed–accuracy trade-off. The studies employed various VR systems, including maze-solving tasks using computer keyboards [34] and smartphones [30], motion capture systems [32,33,35,36], and computerized reciprocal aiming tasks with a computer mouse [31]. One study investigated the effect of VR training on the functional abilities in boys with DMD [36].

### 3.3. Motor Learning

In 2020, da Silva studied the speed–accuracy trade-off among DMD and TD individuals in a computer task (Fitts’ Reciprocal Aiming Task), revealing that DMD individuals took longer, especially for quicker tasks, suggesting that speed-demanding tasks should be prioritized in rehabilitation [31]. De Freitas et al. (2019) compared performances of DMD individuals in a virtual task using different interfaces, including a touch screen, Microsoft Kinect, and Leap Motion. Results indicated significant benefits when using Leap Motion due to its advantages in task acquisition and retention [32]. Quadrado et al. (2019) studied performance in a task using physical and virtual interfaces, with DMD and TD groups showing improved performance with practice and maintained performance during retention. The study also found facilitated transfer from virtual to real environments for DMD individuals when performing a specific task [33]. Capelini et al. (2017) analyzed motor learning disparities using a smartphone game (Marble Maze Classic^®^), finding that practice resulted in improved performance for both DMD and TD groups, which persisted during the retention and transfer phases, showing similar learning patterns [30]. Malheiros et al. (2016) utilized a computer maze task with a keyboard, observing enhanced performance in DMD individuals after practice, but notable differences in movement time between the DMD and control groups [34]. Lastly, Massetti et al. (2018) examined if practicing a task in a virtual environment could improve performance and facilitate transference to a real environment. While performance, short-term retention, and task transfer improved, there was no evidence of transfer between the virtual and real environments [35].

### 3.4. Effectiveness of VR Intervention

A single study investigated the efficacy of incorporating one hundred 15 min sessions of virtual reality gaming using a PlayStation 2 console and motion capture device (EyeToy), alongside dynamic arm support Gainboy^®^, as an adjunct to standard treatments like physical therapy and corticosteroids [36]. The intervention demonstrated significant improvements in muscle strength and elbow range of motion. However, it did not yield any significant enhancements in the Performance of the Upper Limb (PUL) scale.

### 3.5. Risk of Bias

Table 2 presents a summary of the methodological quality of the included studies, indicating the PEDro score and the IVS score. The mean PEDro score of all studies was 5.9 (SD = 1.4, range: 5–7) out of 10. Based on the additional internal validity score (IVS), which considered seven specific PEDro items, out of the seven analyzed publications, two publications (29%) obtained a score classifying the quality of the publication as ‘moderate’, and five (71%) as ‘limited’. Analyzing the individual items of the PEDro questionnaire, it was observed that the publications most frequently lost points for a failure to refer to the blinding of the therapists (100%), the participants (100%), and the assessors (96%). These aspects of blinding were commonly not adequately addressed in the included studies, which may have introduced bias into their results. Furthermore, deductions were incurred due to non-compliance with the ‘concealed allocation’ criterion (71%).

### 3.6. Meta-Analysis

A comprehensive meta-analysis of the effectiveness of VR intervention in comparison to usual care, VR combined with motor treatment, no intervention, or placebo control was regrettably unfeasible due to the dearth of available interventional studies meeting the necessary inclusion criteria. The paucity of research that directly compares these different treatment modalities in controlled settings has precluded the synthesis of a meta-analysis. Only the study conducted by Heutinck et al. compared games with motion capture and gravity compensation for the arms against usual care [36]. The absence of a sufficient number of interventional studies addressing the specific comparisons of interest has hindered the ability to perform a systematic analysis that would yield statistically significant and clinically relevant insights. While virtual reality holds promise as a therapeutic tool, particularly in conjunction with motor treatment, the lack of robust empirical evidence derived from randomized controlled trials has constrained the capacity to draw generalized conclusions.

## 4. Discussion

The primary aim of the study was to provide a comprehensive summary of the current understanding regarding the application of virtual reality in supporting muscular dystrophy rehabilitation. Due to the absence of interventional studies specifically aimed at evaluating the effectiveness of VR application in improving motor performance, the quantitative summary could not be realized.

However, included studies suggest promising outcomes for implementing VR interventions in DMD rehabilitation, showing potential in enhancing motor learning, particularly in the retention and transfer phases. Moreover, individuals with DMD demonstrated learning patterns similar to typically developing individuals [30,32,33]. While individuals with DMD demonstrated improved performance in a computer motor task, their functional performance (time of movement) remained impaired compared to TD individuals [30,31,32,34]. The observed enhancements in motor skill retention with VR interventions for individuals with DMD are significant. These skills directly impact functional independence and overall progress, particularly given the challenges that traditional rehabilitation methods often encounter in achieving lasting retention [37]. VR-based interventions, due to their dynamic and semi-immersive and immersive environments, may offer a more effective approach for the retention phase of motor learning in DMD rehabilitation by enabling repetitive, task-specific training [38]. Considering the progressive nature of DMD, regular and engaging exercises using VR could encourage sustained practice, which may lead to an improvement in physical functionality and, consequently, promote greater independence and an improved quality of life for patients [17]. Exergaming with motion capture, using commercially available systems like PlayStation 4 Camera or Xbox Kinect, offers potential as an engaging, innovative approach for DMD rehabilitation [39]. This was confirmed by a single study included in this review that examined the efficacy of a novel intervention involving 100 short (15-min) sessions of virtual reality gaming utilizing a PlayStation 2 console [36]. While the intervention resulted in notable improvements in muscle strength and elbow range of motion, it did not show statistically significant enhancements in the Performance of the Upper Limb (PUL) assessment. Furthermore, VR interventions may reduce the need for frequent in-person therapy sessions, thereby enabling remote rehabilitation and promoting more accessible and sustainable training.

However, there remains a discrepancy concerning the validation of motor learning transfer between real-world and virtual environments. In the study conducted by Quadrado et al. (2019), individuals with DMD showed improved transfer from a virtual environment to a real environment when performing a coincidence timing task, contradicting the results reported by Massetti et al. (2018) and De Freitas et al. (2019) [30,32,33].

### 4.1. Clinical Implication and Future Study Directions

The studies collectively indicate promising outcomes for clinical implementation of VR interventions in DMD rehabilitation, demonstrating potential in enhancing motor learning. Tasks that emphasize speed and accuracy in VR-based rehabilitation may be particularly beneficial in improving motor abilities and increasing independence for affected individuals. Additionally, the use of no-contact VR systems, such as the Leap Motion interface, demonstrates advantages in acquiring and retaining task performance. Moreover, VR-based training can lead to performance improvements during the acquisition phase, and shows potential for long-term retention and transfer of skills to real-world settings. These findings support the integration of VR technology into rehabilitation programs to address motor deficits and enhance the overall quality of life for individuals with muscular dystrophy. However, among the included studies, only one RCT specifically examined the effectiveness of VR exergames for upper extremity motor rehabilitation, utilizing a limited sample size of 16 individuals with an outdated PlayStation 2 exergaming. Although the study’s primary outcome measure (i.e., PUL) did not show significant changes with VR training, there were indications suggesting that such training might help mitigate the loss of range of motion and strength. Further research is needed to optimize the clinical implementation of VR-based interventions and explore their transferability to real-world environments. It is also advisable to explore various types of muscular dystrophy and incorporate more novel VR systems, including exergaming with commercially available systems like Nintendo Switch, PlayStation 4 Camera, or the older Xbox Kinect. Immersive head-mounted display VR games like Beat Saber could also be considered, as they may be more suitable than conventional 2D screens for training 3D movements in VR-based therapy [40].

### 4.2. Study Limitation

The study has several limitations that should be acknowledged. Firstly, all of the included studies focused exclusively on DMD, and no investigations were conducted on other types of dystrophies, limiting the generalizability of the findings to broader dystrophy populations. Secondly, out of the seven included studies, only one specifically investigated the effectiveness of VR for motor rehabilitation, while the remaining six primarily focused on motor learning. As a result, quantitative meta-analysis to assess the overall effectiveness was not feasible, and the analysis was limited to qualitative synthesis. Furthermore, the diversity in the utilization of VR systems among the included studies adds another limitation. Four studies employed motion capture technology, while the other studies employed different VR systems, making it challenging to directly compare the outcomes, and limiting the generalizability of the results to the wider population.

## 5. Conclusions

The studies investigating the use of VR for muscular dystrophy rehabilitation consistently demonstrated its potential as a valuable intervention. VR-based training proved to be effective in enhancing the motor learning of individuals with Duchenne muscular dystrophy. Furthermore, utilizing motion capture interfaces like Leap Motion in VR scenarios showcased significant advantages, while the practice of visual motor tasks in VR positively impacted performance retention and transfer to real-world settings. These findings highlight the importance of incorporating VR-based interventions in rehabilitation programs to improve the motor abilities and independence for individuals with muscular dystrophy.

## Figures and Tables

**Table 1:** Characteristics of included studies.

Author (Year)	Study Design	*N*	Group Characteristics (Mean Age; tMFM)	Interventions	Outcomes	Conclusions
**Motor learning**						
da Silva et al. (2020) [31]	Cross-sectional study	G1: 17 G2: 17	G1: DMD (15.4 years; tMFM: 46.6) G2: TD (15.4 years)	Computerized Discrete Aiming Task (v.1.0) (total 12 trials)	Movement time, Vignos scale	Tasks that require accuracy should predominately be used in DMD daily activities in order to keep them engaged in social participation.
De Freitas et al. (2019) [32]	RCT	G1: 60 G2: 60	G1: DMD (16.0 years; tMFM: 54.4) G2: TD (16.0 years)	Upper extremity dexterity computer game using 3 different conditions: Leap Motion, Kinect and Touch Screen	Vignos scale, Acquisition, Retention, Transfer	A device with no contact (Leap Motion) facilitated the successful implementation of the proposed task. Therefore, an improvement in performance when using a virtual interface requiring no physical contact for individuals with DMD.
Massetti et al. (2018) [35]	RCT with crossover	G1: 11 G2: 11	G1: DMD (14.8 years; tMFM: 49.8) G2: DMD (16.8 years; tMFM: 55.7)	G1: Virtual task with MoVER software and Kinect sensor G2: Real task with Kinect sensor (total 40 trials)	MFM, Movement time, Acquisition, Retention, Transfer	Both virtual and real tasks promoted improvement of performance, although performance of participants in the real task was better than that in the virtual one.
Capelini et al. (2017) [30]	Two arm study	G1: 50 G2: 50	G1: DMD (17.2 years; tMFM: 48.5) G2: TD (17.3 years)	Moving a virtual ball in virtual maze in smartphone game (total 45 trials)	EK, Vignos scale, Acquisition, Retention Transfer	Practice of a visual motor task in mobile game promoted improvement in performance during the acquisition of the game in groups with DMD and TD.
Quadrado et al. (2019) [33]	Two arm study	G1: 32 G2: 32	G1: DMD (18.0 years tMFM: NS) G2: TD (18.0 years)	Upper extremity dexterity computer game with motion capture device and computer keyboard (total 35 trials)	Movement time, Acquisition, Retention, Transfer	Individuals with DMD, conducting a coincidence timing task in a virtual environment facilitated transfer to the real environment.
Malheiros et al. (2016) [34]	Two arm study	G1: 42 G2: 42	G1: DMD (18.1 years; tMFM: 43.2) G2: TD (18.1 years)	Virtual maze in computer game (total 30 trials)	Movement time, Acquisition, Retention, Transfer	Intervention improved in computational task performance among participants with DMD following practice. Difference in movement time was observed in all attempts among individuals from both groups.
**VR intervention efficacy**						
Heutinck et al. (2018) [36]	explorative RCT	G1: 7 G2: 9	G1: DMD (12.9 years; tMFM: NS) G2: DMD (12.6 years tMFM: NS)	G1: Games with motion capture and gravity compensation for the arms (five 15 min sessions a week for 20 weeks) G2: Usual care	PUL, QMUS, A6MCT, MFM, Global Health Question, Kidscreen-52	Study did not show a significant effect of training on the primary outcome measure, and there were indications that training may decline the loss of range of motion and strength.

G1: group 1; G2: group 2; DMD: Duchenne Muscular Dystrophy; TD: typically developed; NS: not specified; tMFM: total Motor Function Measure scale; PUL: Performance of the Upper Limb; QMUS: quantitative muscle ultrasound; A6MCT: Assisted Six-Minute Cycle Test; EK: Egen Klassifikation scale.

**Table 2:** Methodological quality of the included studies. X represent one out of 10 points assigned.

Author (Year)	(1) *	(2)	(3)	(4)	(5)	(6)	(7)	(8)	(9)	(10)	(11)	Tot.	IVS
da Silva et al. (2020) [31]	X			X				X	X	X	X	5/10	2/7
De Freitas et al. (2019) [32]	X	X		X				X	X	X	X	6/10	3/7
Heutinck et al. (2018) [36]		X		X			X			X	X	5/10	2/7
Massetti et al. (2018) [35]	X	X	X	X				X	X	X	X	7/10	4/7
Capelini et al. (2017) [30]	X	X		X				X	X	X	X	6/10	3/7
Quadrado et al. (2019) [33]	X	X	X	X				X	X	X	X	7/10	4/7
Malheiros et al. (2016) [34]	X			X				X	X	X	X	5/10	2/7
%, X	86	71	29	100	0	0	14	86	86	100	100		

(1) Eligibility criteria. (2) Random allocation. (3) Concealed allocation. (4) Baseline comparability; (5) Blind participants. (6) Blind therapists. (7) Blind assessors. (8) Adequate follow-up. (9) Intention-to-treat analysis. (10) Between-group comparisons. (11) Point estimates and variability. * Eligibility criteria item does not contribute to total PEDro score. IVS: internal validity score.

## Data Availability

Data are available upon reasonable request to the corresponding author.

## Supplementary Materials

The following supporting information can be downloaded at: https://www.mdpi.com/article/10.3390/life14070790/s1.
