# Supplementary material for: A Systematic Review on the Application of Virtual Reality for Muscular Dystrophy Rehabilitation: Motor Learning Benefits
Source: Life (Basel). 2024 Jun 22;14(7):790. doi: 10.3390/life14070790 (PMC11278510; doi:10.3390/life14070790)
Supplement: Supplementary file 1 [file life-14-00790-s001.zip › life-2984959-supplementary.pdf]

Supplementary files – search strategy

PubMed: (("Muscular Dystrophies"[MeSH Terms] OR "muscular dystrophy, duchenne"[MeSH Terms] OR "Myotonic Dystrophy"[MeSH Terms] OR "md1" OR "duchenne muscular dystrophy" OR "duchenne dystrophy" OR "DMD" OR "Becker Muscular Dystrophy" OR "Becker Dystrophy" OR "Limb-Girdle Muscular Dystrophy" OR "Limb-Girdle Dystrophy" OR "Facioscapulohumeral Dystrophy" OR "Facioscapulohumeral Muscular Dystrophy" OR "Emery–Dreifuss Muscular Dystrophy" OR "Emery–Dreifuss Dystrophy" OR "Oculopharyngeal Muscular Dystrophy" OR "Oculopharyngeal Dystrophy" OR "Distal Muscular Dystrophy" OR "Distal Dystrophy" OR "Congenital Dystrophy" OR "Congenital Muscular Dystrophy") AND ("VR" OR "Virtual reality"[MeSH Terms] OR "exergaming"[MeSH Terms] OR "video games"[MeSH Terms] OR "immersion"[MeSH Terms] OR "augmented reality"[MeSH Terms] OR "games, experimental"[MeSH Terms] OR "exergam*" OR "gamercising" OR "Mixed Reality" OR "virtual reality gam*" OR "computer task*" OR "game*"OR "gaming" OR "virtual training" OR "Virtual Reality Exposure Therapy"[Mesh] OR "game-based rehabilitation" OR "head mounted display" OR "Nintendo" OR "WiiPD" OR "WII fit" OR "Wii-hab" OR "Xbox" OR "PlayStation" OR "Kinect" OR "VR" OR "Virtual Reality Exercis*" OR "Active Video Gam*" OR "Serious gam*" OR "augmented reality" OR "computer")) AND ("gait"[MeSH Terms] OR "Postural Balance"[MeSH Terms] OR "Motor skills"[MeSH Terms] "Muscle strength"[MeSH Terms] OR "Muscle contraction"[MeSH Terms] OR "Muscle Fatigue"[MeSH Terms] OR "gait analysis"[MeSH Terms] OR "Activities of Daily Living"[Mesh] OR "Mobility Limitation"[Mesh] OR "Muscular strength" OR "Walk Test"[Mesh] OR "motor function" OR "ADL" OR "strength" OR "motor learning" OR "motor impairment" OR "motor performance" OR "performance")

SCOPUS: TITLE-ABS-KEY("Muscular Dystrophy" OR "Muscular Dystrophies" OR "duchenne muscular dystrophy" OR "DMD" OR "duchenne dystrophy" OR "Myotonic Dystrophy" OR "md1" OR "Becker Muscular Dystrophy" OR "Becker Dystrophy" OR "Limb-Girdle Muscular Dystrophy" OR "Limb-Girdle Dystrophy" OR "Facioscapulohumeral Dystrophy" OR "Facioscapulohumeral Muscular Dystrophy" OR "Emery-Dreifuss Muscular Dystrophy" OR "Emery-Dreifuss Dystrophy" OR "Oculopharyngeal Muscular Dystrophy" OR "Oculopharyngeal Dystrophy" OR "Distal Muscular Dystrophy" OR "Distal Dystrophy" OR "Congenital Dystrophy" OR "Congenital Muscular Dystrophy") AND TITLE-ABS-KEY("VR" OR "Virtual reality" OR "exergaming" OR "video games" OR "immersion" OR "augmented reality" OR "experimental games" OR "exergam*" OR "gamercising" OR "Mixed Reality" OR "virtual reality gam*" OR "computer task*" OR "game*" OR "gaming" OR "virtual training" OR "Virtual Reality Exposure Therapy" OR "game-based rehabilitation" OR "head mounted display" OR "Nintendo" OR "WiiPD" OR "WII fit" OR "Wii-hab" OR "Xbox" OR "PlayStation" OR "Kinect" OR "VR" OR "Virtual Reality Exercis*" OR "Active Video Gam*" OR "Serious gam*" OR "augmented reality" OR "computer") AND TITLE-ABS-KEY("gait" OR "Postural Balance" OR "Motor skills" "Muscle strength" OR "Muscle contraction" OR "Muscle Fatigue" OR "gait analysis" OR "Activities of Daily Living" OR "Mobility Limitation" OR "Muscular strength" OR "Walk Test" OR "motor function" OR "ADL" OR "strength" OR "motor learning" OR "motor impairment" OR "motor performance" OR "performance")

Web of Science: ((ALL=("Muscular Dystrophy" OR "Muscular Dystrophies" OR "duchenne muscular dystrophy" OR "DMD" OR "duchenne dystrophy" OR "Myotonic Dystrophy" OR "md1" OR "Becker Muscular Dystrophy" OR "Becker Dystrophy" OR "Limb-Girdle Muscular Dystrophy" OR "Limb-Girdle Dystrophy" OR "Facioscapulohumeral Dystrophy" OR "Facioscapulohumeral Muscular Dystrophy" OR "Emery–Dreifuss Muscular Dystrophy" OR "Emery–Dreifuss Dystrophy" OR "Oculopharyngeal Muscular Dystrophy" OR "Oculopharyngeal Dystrophy" OR "Distal Muscular Dystrophy" OR "Distal Dystrophy" OR "Congenital Dystrophy" OR "Congenital Muscular Dystrophy")) AND ALL=("VR" OR "Virtual reality" OR "exergaming" OR "video games" OR

"immersion" OR "augmented reality" OR "experimental games" OR "exergam*" OR "gamercising" OR "Mixed Reality" OR "virtual reality gam*" OR "computer task*" OR "game*" OR "gaming" OR "virtual training" OR "Virtual Reality Exposure Therapy" OR "game-based rehabilitation" OR "head mounted display" OR "Nintendo" OR "WiiPD" OR "WII fit" OR "Wii-hab" OR "Xbox" OR "PlayStation" OR "Kinect" OR "VR" OR "Virtual Reality Exercis*" OR "Active Video Gam*" OR "Serious gam*" OR "augmented reality" OR "computer")) AND ALL=("gait" OR "Postural Balance" OR "Motor skills" "Muscle strength" OR "Muscle contraction" OR "Muscle Fatigue" OR "gait analysis" OR "Activities of Daily Living" OR "Mobility Limitation" OR "Muscular strength" OR "Walk Test" OR "motor function" OR "ADL" OR "strength" OR "motor learning" OR "motor impairment" OR "motor performance" OR "performance")

Cochrane library

#1	"Muscular Dystrophy" OR "Muscular Dystrophies" OR "duchenne muscular dystrophy" OR "DMD" OR "duchenne dystrophy" OR "Myotonic Dystrophy" OR "md1" OR "Becker Muscular Dystrophy" OR "Becker Dystrophy" OR "Limb-Girdle Muscular Dystrophy" OR "Limb-Girdle Dystrophy" OR "Facioscapulohumeral Dystrophy" OR "Facioscapulohumeral Muscular Dystrophy" OR "Emery–Dreifuss Muscular Dystrophy" OR "Emery–Dreifuss Dystrophy" OR "Oculopharyngeal Muscular Dystrophy" OR "Oculopharyngeal Dystrophy" OR "Distal Muscular Dystrophy" OR "Distal Dystrophy" OR "Congenital Dystrophy" OR "Congenital Muscular Dystrophy"	1670
#2	"VR" OR "Virtual reality" OR "exergaming" OR "video games" OR "immersion" OR "augmented reality" OR "experimental games" OR "NEXT exergam*" OR "gamercising" OR "Mixed Reality" OR "virtual reality NEXT gam*" OR "computer NEXT task*" OR "NEXT game*" OR "gaming" OR "virtual training" OR "Virtual Reality Exposure Therapy" OR "game-based rehabilitation" OR "head mounted display" OR "Nintendo" OR "WiiPD" OR "WII fit" OR "Wii-hab" OR "Xbox" OR "PlayStation" OR "Kinect" OR "VR" OR "Virtual Reality NEXT Exercis*" OR "Active Video NEXT Gam*" OR "Serious NEXT gam*" OR "augmented reality" OR "computer"	67444
#3	"gait" OR "Postural Balance" OR "Motor skills" "Muscle strength" OR "Muscle contraction" OR "Muscle Fatigue" OR "gait analysis" OR "Activities of Daily Living" OR "Mobility Limitation" OR "Muscular strength" OR "Walk Test" OR "motor function" OR "ADL" OR "strength" OR "motor learning" OR "motor impairment" OR "motor performance" OR "performance"	200479
#4	#1 AND #2 AND #3	62 (29 trials)
